# Mendelian randomization of chronic hepatitis B and cardiovascular disease

**DOI:** 10.3389/fcvm.2024.1332557

**Published:** 2024-03-15

**Authors:** Dongjie Wu, Feiyang Xiong, Qingzhi Ran, Jing Liu, Qingjuan Wu, Liang Wang, Wenliang Lv

**Affiliations:** ^1^ Department of Infectious Diseases, Guang'anmen Hospital, China Academy of Chinese Medicine Sciences, Beijing, China; ^2^ Hunan University of Traditional Chinese Medicine, Changsha, Hunan, China; ^3^ Department of Cardiovascular Diseases, Guang'anmen Hospital, China Academy of Chinese Medicine Sciences, Beijing, China; ^4^ Beijing Century Forum Hospital, Capital Medical University, Beijing, China

**Keywords:** chronic hepatitis B (CHB), cardiovascular disease, Mendelian randomization (MR), causality, genome-wide association studies (GWAS), atherosclerosis, ischemic stroke (IS)

## Abstract

**Background:**

Evidence from observational studies suggests that chronic hepatitis B (CHB) is associated with cardiovascular disease (CVD). However, results have been inconsistent and causality remains to be established. We utilized two-sample Mendelian randomization (MR) to investigate potential causal associations between CHB and CVD, including atherosclerosis, coronary heart disease, hypertension, and ischemic stroke.

**Methods:**

The analysis was conducted through genome-wide association studies (GWAS), considering chronic hepatitis B as the exposure and cardiovascular disease as the endpoint. The primary method for evaluating causality in this analysis was the inverse-variance weighted (IVW) technique. Additionally, we employed the weighted median, MR-Egger regression, weighted mode, and simple mode methods for supplementary analyses. Finally, heterogeneity tests, sensitivity analyses, and multiple effects analyses were conducted.

**Results:**

In a random-effects IVW analysis, we found that genetic susceptibility to chronic hepatitis B was associated with an increased risk of atherosclerosis [OR = 1.048, 95% CI (1.022–1.075), *P* = 3.08E-04], as well as an increased risk of coronary heart disease [OR = 1.039, 95% CI (1.006–1.072), *P* = 0.020]. However, it was found to be inversely correlated with ischemic stroke risk [OR = 0.972, 95% CI (0.957–0.988), *P* = 4.13E-04]. There was no evidence that chronic hepatitis B was associated with hypertension [OR = 1.021, 95% CI (0.994–1.049), *P* = 0.121].

**Conclusion:**

Our research indicates that chronic hepatitis B has a correlation with an elevated risk of developing atherosclerosis and coronary heart disease, while it is associated with a decreased risk of experiencing an ischemic stroke.

## 1 Introduction

Cardiovascular disease (CVD) encompasses a variety of ailments impacting the heart and circulatory system, which includes conditions such as atherosclerosis, coronary artery disease (CAD), high blood pressure, ischemic stroke (IS), cerebral infarction (CI), cardiac arrhythmia (CA), and cardiac insufficiency (CI). According to statistics, the number of deaths due to cardiovascular disease is close to 190,000 globally as of 2020, an increase of 7.201% from 2018 (1–3). Mortality and prevalence of cardiovascular diseases vary significantly by region. Compared with the low CVD mortality rates in North America and Western Europe, the rates are highest in Eastern Europe and Central Asia. Moreover, the incidence of cardiovascular disease in North Africa and the Middle East is notably elevated (2). This is not only a huge health toll on humanity, but also a heavy socio-economic burden (4, 5). The onset and progression of CVD frequently result from the complex interplay among genetic factors, environmental triggers, and immune dysregulation (6, 7).

The worldwide prevalence of chronic hepatitis B virus (HBV) infection is notably substantial, impacting over two billion individuals around the globe. Among this group, there are around 350 million persons who are persistent carriers of the virus (8). Numerous investigations indicate a heightened occurrence of CVD in individuals diagnosed with CHB (9). For instance, a study utilizing a cross-sectional cohort design determined a substantial correlation between chronic hepatitis B (CHB) and the incidence of carotid atherosclerosis, with an Odds Ratio (OR) of 1.57 and a 95% Confidence Interval (CI) ranging from 1.10 to 2.24, *P*-value less than 0.05 (10). Another meta-analysis also showed a significant association between CHB and CHD (11). However, there are some studies that deny the relationship between CHB and CVD risk. A cross-sectional study in Taiwan did not find a correlation between HBV seropositivity and severity of carotid atherosclerosis (12). One cohort study even showed that HBV infection was associated with a reduced risk of CHD [OR = 0.81, 95% CI (0.67–0.98)] (13). Yet, a separate meta-analysis did not substantiate a notable link between patients infected with HBV and the incidence of CHD (14). In a case-control study from Taiwan, it was observed that patients with diabetes mellitus who also had chronic HBV infection showed a reduced risk for ischemic stroke, heart failure, and overall mortality, which is consistent with the findings of our study (15).

The current findings are inconclusive as to whether CHB directly contributes to CVD risk due to insufficient sample size and potential confounding factors. Further clinical investigations are warranted to delve deeper into this correlation. Nonetheless, there is a scarcity of research on the incidence of CVD and related risk factors among individuals with chronic hepatitis B.

In epidemiology, the utilization of Mendelian randomization (MR) analysis has gained significant prominence in recent years as a powerful tool for causal inference studies. The fundamental principle of MR studies is that the random allocation of alleles during gamete formation across parental generations simulates a randomized grouping of populations, similar to the design of randomized controlled trials (16). By genetic variants being fixed at birth and persisting throughout an individual's life, MR studies mitigate the potential for reverse causation. Furthermore, since genetic variation remains unaffected by subsequent environmental and behavioral factors, MR studies offer enhanced accuracy in assessing causality (17). By employing genetic variation as instrumental variables (IVs), researchers are able to effectively account for confounding variables in a study, thereby controlling for the influence of extraneous factors (18). Progress in GWAS has produced a substantial amount of reliable and trustworthy IVs for MR analysis. In light of recent progress, our research employed a dual-sample Mendelian Randomization strategy to explore the potential link between genetic indicators of CHB and the risk factors for CVD.

## 2 Materials and methods

### 2.1 Study design

**Figure 1:**
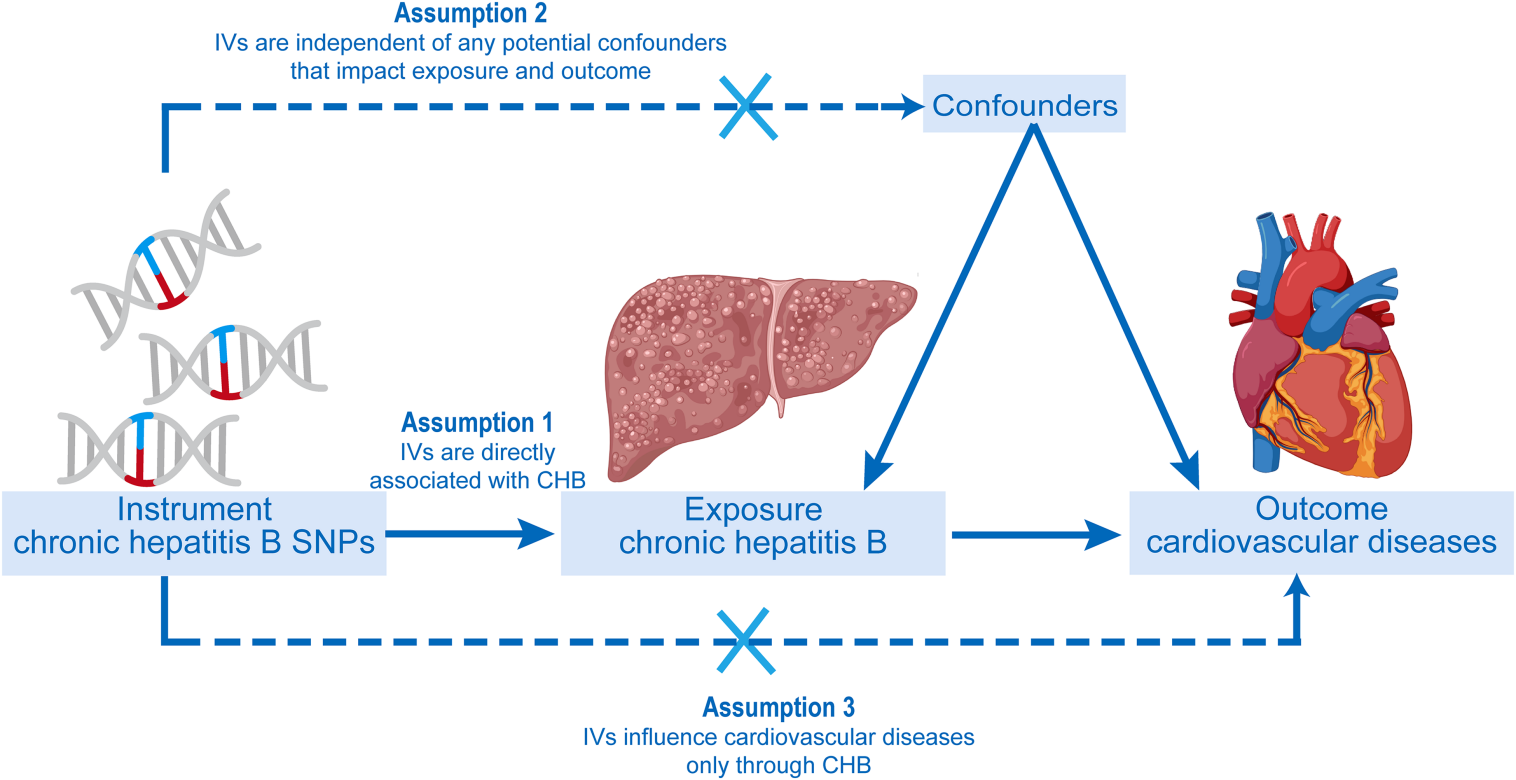
The progression of the two-sample Mendelian randomization (MR) study and its three principal postulates.

A two-sample MR study was conducted to investigate the causal relationship between CHB and CVD. The inverse variance weighting (IVW) approach was primarily used to make causal conclusions about the effect of CHB on the development of CVD. The fundamental principle of MR involves the use of genetic variants associated with exposure (CHB) and outcome (CVD) as IVs to infer causality. In our study, CHB was the exposure interest, and single nucleotide polymorphisms (SNPs) with significant ties to CHB were utilized as instrumental variables (IVs), targeting various cardiovascular diseases as the outcome measures. The MR analysis was conducted under three fundamental presumptions. A schematic of the trio of principal MR postulates and the research framework is depicted in Figure 1 (19, 20).

### 2.2 Data sources

The dataset for the exposure and outcome variables was independently retrieved from the GWAS portal at https://gwas.mrcieu.ac.uk/. Information pertaining to CHB and ischemic stroke (IS) was obtained from the EBI GWAS catalog (21). Regarding the outcome variables such as atherosclerosis, coronary heart disease, and hypertension, the GWAS data were sourced from the FinnGen database (https://www.finngen.fi/fi). The datasets employed in this analysis were concentrated on European demographics, with further specifics accessible in Table 1. The CHB GWAS summary statistic data was obtained from a published study consist of 351,885 European participants, of which there were 145 participants who were diagnosed of CHB in UK Biobank, according to ICD10 code (B18.0/B18.1), and phecode (070.2). In terms of the outcome GWAS data, except for IS, which was obtained from published study consist of 484,121 European individuals, the others including Atherosclerosis, CHD, and hypertension were obtained from FinnGen consist of 234,566, 218,792, and 218,754 Finnish individuals respectively. The CHB GWAS dataset and GWAS datasets of CVD originated from different consortiums to decrease the potential bias caused by sample overlap. In addition, all GWAS datasets involved in this study included populations of European ancestry to mitigate bias from population stratification.

**Table 1:** Information on data from Mendelian randomization analysis.

Traits	Dataset	Cases	Controls	Sample size	Population	Year
CHB	ebi-a-GCST90018804	145	351,740	351,885	European	2021
Atherosclerosis	Finn-b-I9_CORATHER	23,363	187,840	234,566	European	2021
CHD	Finn-b-I9_CHD	21,012	197,780	218,792	European	2021
Hypertension	Finn-b-I9_HYPTENS	55,917	162,837	218,754	European	2021
IS	ebi-a-GCST90018864	11,929	472,192	484,121	European	2021

### 2.3 IVs selection and evaluation

Initially, significant SNPs associated with the exposure variable were extracted as IVs from the IEU Open GWAS database. A stringent filtering criterion of *P* < 5 × 10^−8^ was applied to the selection process. To ensure independence among the IVs, we excluded those in linkage disequilibrium (LD) by setting the parameters *r*^2 ^< 0.01 and kb = 1,000. When the quantity of SNPs screened was found to be inadequate, we adjusted the threshold parameters to a *P*-value smaller than 5 × 10^−6^. Additionally, SNPs with palindromic structures were excluded by correcting the data for exposure and outcome in the same direction. To evaluate the potential bias from weak instruments, we calculated the F statistic for each SNP using the formula *F = β^2^_exposure_/SE^2^_exposure_*. IVs with an F statistic exceeding 10 were selected to lessen the likelihood of bias stemming from weak instruments (22). Supplementary Tables 1–S4 provides a detailed overview of these IVs.

### 2.4 Mendelian randomization analyses

For the purpose of this study, five distinct methods were utilized to estimate the causal associations between genetic variations associated with CHB and CVD (23). In our Mendelian Randomization study, the IVW approach, which aggregates genotype data, served as the primary technique to merge the Wald ratios for each SNP through a meta-analytical process to derive the comprehensive estimate (24, 25). The estimated outcome, when the intercept is fixed at zero, is depicted by the incline of the weighted regression correlating the effect of the outcome with the effect of the exposure. To compensate for the limitation of IVW, which assumes all genetic variables to be valid IVs, we employed the weighted median method. This approach allowed us to amalgamate data from multiple genetic variables into a single consistent causal assessment, even if up to 50% of the information originated from potentially invalid IVs (26, 27). In this investigation, the MR-Egger regression method was utilized to evaluate the potential pleiotropic effects of all SNPs. This method not only offers the capability to detect horizontal heterogeneity through intercept tests but also provides estimates after adjusting for any pleiotropic effects (28). In the sensitivity analysis framework, both the simple model and the weighted model approaches serve as supplementary MR techniques and ought to be utilized alongside other methods. The adoption of various methods, each predicated on different assumptions, as opposed to exclusive reliance on a singular method, is a judicious strategy to fortify the integrity of the evaluation outcomes. This approach guarantees a thorough and dependable assessment of the causal links between genetic variants associated with CHB and CVD (29). A causal relationship can be inferred if the primary method, IVW, demonstrates significant results (*P* < 0.05), and the other implemented methods exhibit consistent findings in the same direction as the IVW approach.

### 2.5 Sensitivity analysis

To assess the stability and reliability of Mendelian randomization results, this study implemented several quality control methods. To commence, Cochran's *Q*-test was utilized to evaluate the heterogeneity in the individual genetic variation estimates. Upon obtaining a *p*-value less than 0.05 from Cochran's *Q*-test, which suggests heterogeneity, the final MR analysis was conducted employing the IVW random effects model (30). Secondly, to detect potential pleiotropy, We also used the MR-Egger-intercept test, where a *p*-value greater than 0.05 indicated the absence of horizontal pleiotropy (31). In our study, we utilized the MR-PRESSO (Mendelian Randomization Pleiotropy Residual Sum and Outlier) approach as a third step. This method was employed to identify any potential pleiotropic effects and to remove outlier SNPs that may skew the results. This was then followed by a reanalysis. Fourthly, the Leave-one-out sensitivity analysis was utilized to compute the Mendelian Randomization outcomes by consecutively excluding each instrumental variable. This process ensures the robustness of the MR findings (32). If the estimated MR results significantly differed when an instrumental variable was excluded, it indicates sensitivity to that variable. In the two-sample MR analysis conducted for this investigation, we employed the R programming environment (version 4.3.1), in conjunction with the Two Sample MR package (version 0.5.7). The threshold for statistical significance was established at an alpha level of 0.05.

## 3 Results

### 3.1 Characteristics of the selected SNPs

To pinpoint IVs linked with CHB, we executed an exhaustive exploration within GWAS, setting the bar for significance at a *P*-value less than 5 × 10^−8^. To account for any linkage disequilibrium (LD), we excluded IVs with an *r*^2^ value below 0.01 within a range of 1000KB. Furthermore, our study excluded palindromic single nucleotide polymorphisms (SNPs), which are SNPs whose alleles are a nucleotide and its reverse complement. Additionally, we removed palindromic SNPs that had moderate allele frequencies. The final analysis included the remaining screened SNPs, which are reported in Supplementary Tables S1–4. In assessing the robustness of the instrumental variables, the F-statistic was applied, revealing no signs of weak instrument bias as all F-statistic values were above 10.

### 3.2 MR analysis of CHB and CVD

**Figure 2:**
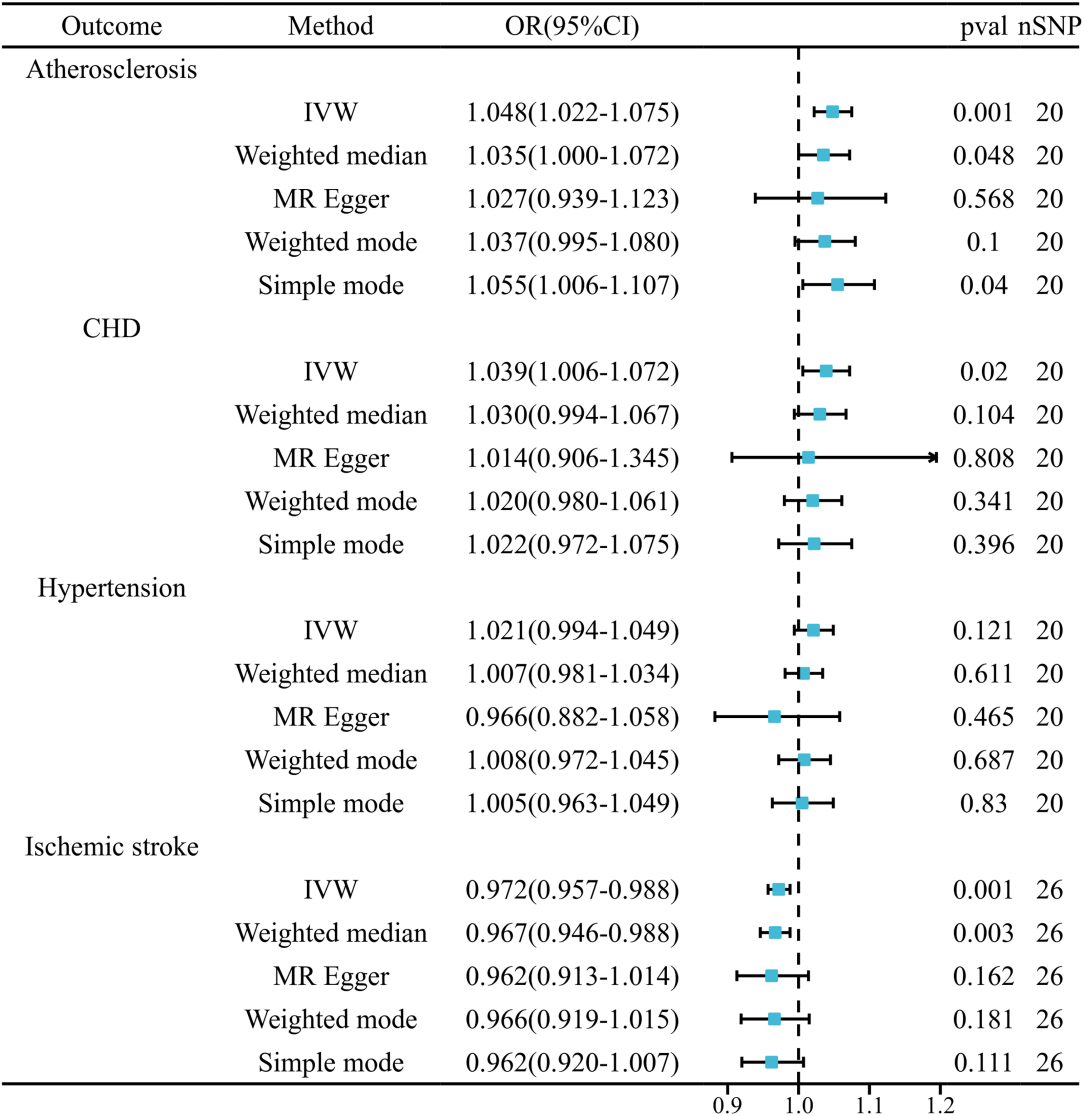
Mendelian randomization estimates of cardiovascular disease risk from chronic hepatitis B.

Figure 2 presents the statistical results obtained from the MR analysis. Employing the random-effects IVW approach, our analysis revealed a significant association between genetic susceptibility to CHB and a heightened risk of developing atherosclerosis and coronary heart disease. Additionally, it was found that individuals with this genetic susceptibility had a reduced risk of IS. Nonetheless, the incidence rate of high blood pressure did not exhibit a notable disparity. Compared to the control group, patients with CHB exhibited a 1.048-fold higher prevalence of atherosclerosis [OR = 1.048, 95% CI (1.022–1.075), *P* = 3.08E-04] and a 1.039-fold higher prevalence of CHD [OR = 1.039, 95% CI (1.006–1.072), *P* = 0.020]. Conversely, the prevalence of IS was 0.972 times lower in CHB patients compared to controls [OR = 0.972, 95% CI (0.957–0.988), *P* = 4.13E-04]. In the comparative analysis of hypertension prevalence, no marked differences were detected between patients with CHB and the control group [OR = 1.021, 95% CI (0.994–1.049), *P* = 0.121]. (Supplementary Figure S1 Scatter plot).

### 3.3 Sensitivity analysis of Mendelian randomization

**Figure 3:**
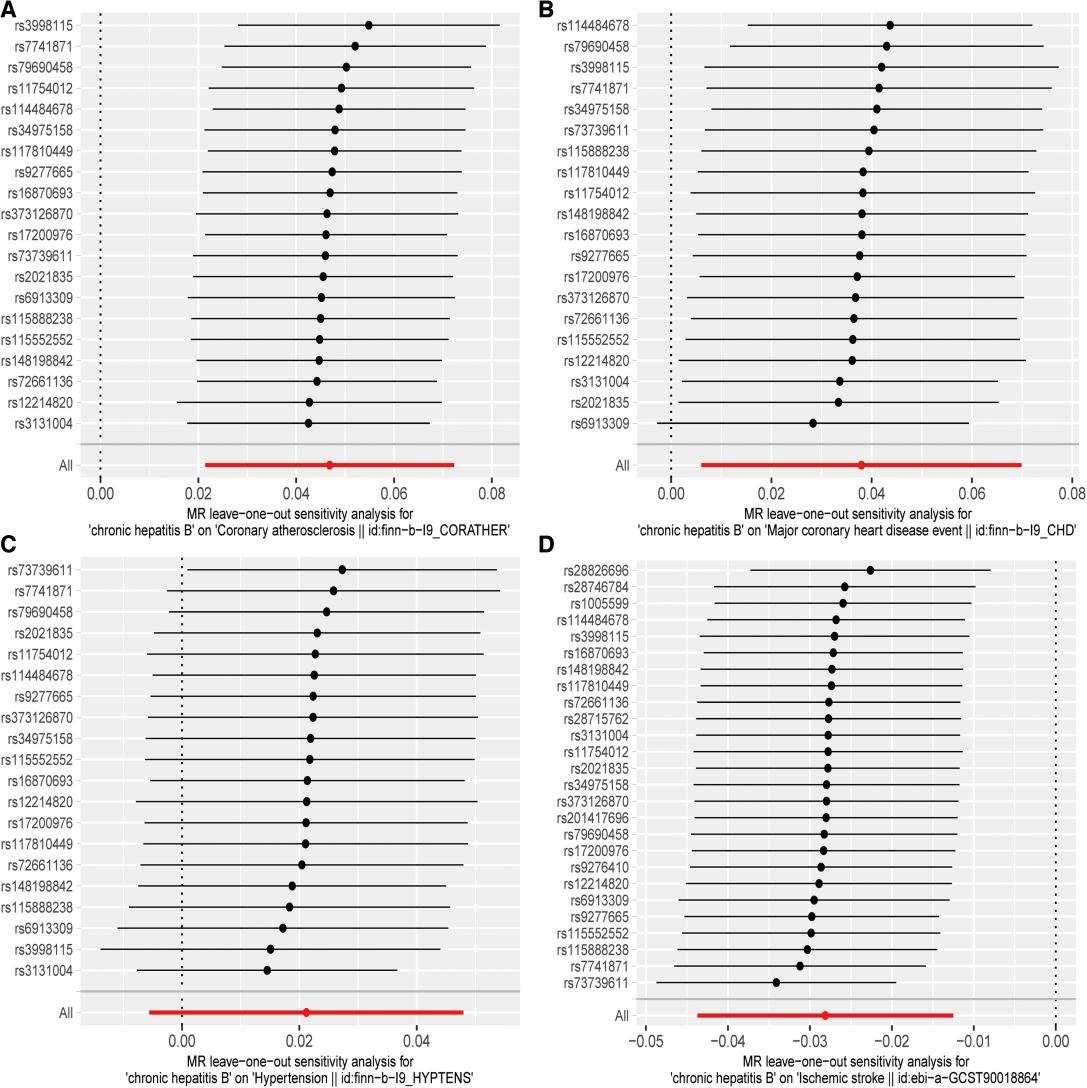
“Leave-one-out” sensitivity analysis results: (**A**) CHB and atherosclerosis; (**B**) CHB and CHD; (**C**) CHB and hypertension; (**D**) CHB and IS.

Initially, the heterogeneity assessment revealed a *p*-value from Cochran's *Q*-statistic that was under 0.05, signifying detectable heterogeneity across the SNPs (Supplementary Figure S2 Funnel plot). In the ensuing MR study, the primary method of analysis was the IVW approach with random effects. Concurrently, the MR-Egger regression's intercept evaluation did not indicate any signs of horizontal pleiotropy in the IVs associated with both CHB and diverse CVDs (more details in Table 2). Furthermore, the Leave-one-out analysis revealed that no SNP exerted a discernible influence on the putative causative link between CHB and CVD risk (refer to Figure 3).

**Table 2:** Tests for multiplicity and heterogeneity of IVs in Mendelian randomization analysis of CHB for CVD.

Outcomes	Heterogeneity test						Pleiotropy test		
	MR-Egger			IVW			MR-Egger		
	Q	Q_df	Q_pval	Q	Q_df	Q_pval	Intercept	SE	*P*
Atherosclerosis	20.51	18	0.30	20.75	19	0.35	0.008	0.018	0.65
CHD	30.54	18	0.03	30.86	19	0.04	0.010	0.023	0.67
Hypertension	39.95	18	0.002	43.47	19	0.001	0.023	0.019	0.22
IS	168.07	19	6.78E-26	168.08	20	2.05E-25	0.001	0.030	0.97

## 4 Discussion

In this study, we conducted the first systematic exploration using MR to investigate the potential causal link between chronic HBV infection and CVD. Our findings indicate that individuals with a genetic predisposition to CHB have an elevated risk of developing atherosclerosis and CHD, while experiencing a lower risk of IS. Nevertheless, no conclusive evidence was discovered to establish a correlation between CHB and the likelihood of developing hypertension.

Chronic HBV infection contributes to numerous liver-related health issues worldwide, including cirrhosis, liver dysfunction, and hepatocellular carcinoma, which is the primary cause of liver disease globally (33–35). Furthermore, patients with CHB have an elevated risk of non-liver-related conditions, such as cardiovascular disease (36). Research indicates that chronic infections may contribute to the development and progression of atherosclerosis, which in turn leads to cardiovascular disease (37). In a cohort study examining cross-sectional data, individuals with chronic HBV infection exhibited an increased incidence of carotid atherosclerosis (10). An additional study provided compelling evidence of a significant association between HBV infection and early atherosclerosis. Crucially, the observed correlation was determined to be autonomous of conventional risk factors, insulin resistance, and elements of the metabolic syndrome assistant (38). Over a 7-year follow-up period, Tseng et al. discovered that the risk of acute ischemic stroke was significantly lower among individuals with both mild and severe HBV infections compared to those in the control group. These findings were based on data obtained from the Taiwan National Health Insurance Program (39). In a similar vein, a comprehensive study involving a large cohort revealed that individuals with HBsAg seropositivity had a lower likelihood of experiencing an ischemic stroke and myocardial infarction. However, it also indicated an elevated risk for hemorrhagic stroke (40). However, it has been challenging to establish a potential causal association between CHB and CVD. This can be attributed to the fact that the majority of studies have not sufficiently addressed the presence of confounding factors. In general, compared to previous studies, our investigation provides evidence of a shared genetic effect between CHB and CVD. We have taken steps to minimize potential confounding effects, reducing the risk of false associations, and our findings suggest a plausible causal link between CHB and CVD.

Although our research suggests a possible link, it is essential to recognize that the mechanisms underlying cardiovascular disease in connection with HBV infection are not yet fully understood. As such, one should be prudent when drawing conclusions from the findings of our study. Several possible mechanisms could explain this association. Hepatitis B virus-induced pro-inflammatory effects (41) and steatosis (42) might contribute to endothelial dysfunction and accelerated atherosclerosis, thus playing a crucial role in the development of cardiovascular disease. Moreover, the development of atherosclerosis associated with HBV has been postulated to be attributed to viral-induced oxidative damage and the presence of a pro-inflammatory state in individuals carrying the chronic HBsAg infection (43). In patients with HBV, there is a noted decrease in elements associated with heightened susceptibility to atherosclerosis (44, 45). This includes a decline in serum levels of Paraoxonase-1 and Arylesterase activities, along with reduced Plasma Free Sulfhydryl Groups and a lower Total Antioxidant Capacity when compared to individuals without infection (46). The observed decrease in the aforementioned factors in HBV-infected individuals may play a role in the development of atherosclerosis. Additionally, it is well-established that CHB patients experience the progression of fibrosis and an augmented immune response (47–49), suggesting that this dysfunctional immune response may also increase cardiovascular risk. Nevertheless, it's important to consider that previous studies suggesting no or negative associations between HBV infection and atherosclerosis may have been influenced by selection bias. Individuals with known chronic liver disease, including those with HBV infection, are more likely to adopt healthier lifestyles and have improved access to healthcare (50). These previous studies may not account for this possibility. In summary, we speculate that the pro-inflammatory effects and augmented immune response induced by Hepatitis B virus may result in endothelial dysfunction and subsequently induce atherosclerosis, and the following CVD.

Regarding the causal relationship between CHB and reduced risk of IS, it has been suggested (39) that the reduced risk of acute ischemic stroke in patients with HBV may be due to abnormal liver function caused by HBV infection, which seems to have a greater effect than that of inflammation-in cerebral atherosclerosis. While some previous literature has discussed a positive correlation between abnormal liver function and ischemic stroke (51, 52), it is important to note that our study found a lower risk of IS in patients with HBV. However, the prevention of IS in HBV patients still warrants attention because previous studies may not have fully accounted for other potential protective factors against cardiovascular disease. Factors such as modification of immunity, physical activity, and HBV medication may all contribute to reducing the risk of IS in HBV patients. Further elucidation of the individual pathophysiologic mechanisms linking HBV infection and various cardiovascular diseases is still needed.

In clinical practice, treatments for different categories of CVD patient are various. For CVD patients combined with CHB, these findings suggest that individualized treatment strategies that target shared genetic variants between CHB and CVD may be more effective. For instance, drug repurposing or new drug development targeting protein coding variants may be possible in the future for the treatment for CVD patients with CHB. CHB-related genetic marker-based risk assessment may help early identification of high-risk of CVD for patients of with CHB. Thus far, there was no causal evidence of anti-viral therapy on clinical outcome among patients with or without CVD, but our study give the causal evidence between CHB and CHD, which will serve as a reminder for subsequent drug-targeted MR studies of anti-viral therapy on various clinical outcomes. In general, our findings are expected to improve precision treatment strategies and early diagnosis for CVD patients with CHB.

Our study possesses several strengths. Firstly, our study conducted a Mendelian randomization analysis, which is the first of its kind, to explore a potential causal connection between CHB and various CVDs. This method enabled us to reduce the effects of confounding variables and the possibility of reverse causality. Secondly, we utilized a large and current GWAS dataset, ensuring no overlap between exposure and outcome, which enhances the reliability of the findings. Thirdly, IVs were thoroughly assessed within each group to minimize instrumental bias. Fourthly, multiple analytical methods were employed and consistently yielded similar results. Moreover, sensitivity analysis, such as MR-PRESSO which is able to identify the potential pleiotropic effects through any potential confounding factors and remove outlier SNPs, further confirm the robustness of our findings.

Nonetheless, it is important to acknowledge the limitations of our study. Primarily, despite our efforts to address multiplicity through various analytical methods, it was not possible to completely mitigate all potential issues related to this aspect. Nevertheless, the consistent findings obtained from these diverse methods provided support to the conclusions drawn in this study and indicated the absence of horizontal multiplicity. Another important consideration is the variation in prevalence and mortality rates of chronic hepatitis B among different racial groups. In our Mendelian randomization analysis, it is pertinent to note that the cohort comprised individuals exclusively with European lineage, which may limit the generalizability of our results to other ethnicities. Therefore, when deducing the potential causative links between chronic hepatitis B and cardiovascular conditions in varied demographics, it is advisable to proceed with prudence. Moreover, the smaller number of individuals diagnosed with CHB may result in fewer genome-side significant variants that GWAS can identify, which could subsequently decrease the number of IVs we can used and the power of our MR analysis. Furthermore, it is crucial to interpret the relatively low odds ratios (ORs) with caution. We look forward to future comprehensive studies that can further investigate this potential relationship in greater depth.

## 5 Conclusion

In conclusion, our research indicates a probable causal link between CHB and the development of atherosclerosis, a heightened predisposition to CHD, and a reduced likelihood of experiencing an ischemic stroke. This study's outcomes enhance the comprehension of the fundamental processes associated with CHB and underscore the importance of thorough cardiovascular disease evaluation and management in patients with CHB. However, the estimates derived from our Mendelian randomization study should be treated with caution because they are relatively small. It is imperative that additional studies be conducted to elucidate the biological processes underlying these observations.

## Data Availability

Publicly available datasets were analyzed in this study. This data can be found here: (i) the IEU Open GWAS project (https://gwas.mrcieu.ac.uk/) and (ii) the FinnGen database (https://www.finngen.fi/fi).
