# Supplementary material for: Mendelian randomization of chronic hepatitis B and cardiovascular disease
Source: Front Cardiovasc Med. 2024 Mar 15;11:1332557. doi: 10.3389/fcvm.2024.1332557 (PMC10978653; doi:10.3389/fcvm.2024.1332557)
Supplement: Supplementary file 1 [file Datasheet1.docx]

Supplementary Material

## Supplementary Table

Supplemental Table 1 Mendelian randomization analysis of chronic hepatitis B and atherosclerosis.

| SNP | Chr | Position | A1 | A2 | EAF | F | Chronic hepatitis B | | | Atherosclerosis | | |
| --- | --- | --- | --- | --- | --- | --- | --- | --- | --- | --- | --- | --- |
|  |  |  |  |  |  |  | **Beta** | **SE** | **P-val** | **Beta** | **SE** | **P-val** |
| rs114484678 | 6 | 32215057 | C | T | 0.063896 | 40.15369 | -0.3143 | 0.0496 | 2.33E-10 | 0.0102 | 0.0255 | 0.690799 |
| rs115552552 | 6 | 33346116 | T | C | 0.039869 | 50.55374 | -0.5553 | 0.0781 | 1.13E-12 | -0.0513 | 0.0332 | 0.1226 |
| rs115888238 | 6 | 32967965 | C | G | 0.077911 | 80.96698 | -0.4904 | 0.0545 | 2.17E-19 | -0.0433 | 0.0294 | 0.1411 |
| rs11754012 | 6 | 33597696 | T | C | 0.138049 | 77.13696 | -0.4892 | 0.0557 | 1.62E-18 | -0.0107 | 0.0206 | 0.604301 |
| rs117810449 | 6 | 32445700 | C | T | 0.054877 | 46.10614 | 0.3171 | 0.0467 | 1.16E-11 | -0.0146 | 0.0381 | 0.7008 |
| rs12214820 | 6 | 27537104 | A | G | 0.123769 | 37.77547 | -0.4499 | 0.0732 | 7.97E-10 | -0.0367 | 0.0171 | 0.03166 |
| rs148198842 | 6 | 30690169 | A | G | 0.024112 | 49.94494 | -0.5576 | 0.0789 | 1.55E-12 | -0.104 | 0.0561 | 0.063779 |
| rs16870693 | 6 | 32711691 | A | C | 0.060517 | 43.00333 | 0.2728 | 0.0416 | 5.55E-11 | -0.1178 | 0.2803 | 0.6742 |
| rs17200976 | 6 | 31674996 | G | A | 0.031991 | 40.89907 | -0.4029 | 0.063 | 1.59E-10 | -0.2003 | 0.122 | 0.1005 |
| rs2021835 | 6 | 27778097 | T | C | 0.039978 | 30.40279 | -0.4571 | 0.0829 | 3.47E-08 | -0.0353 | 0.0276 | 0.2008 |
| rs3131004 | 6 | 31095294 | G | A | 0.568226 | 41.44948 | 0.1925 | 0.0299 | 1.16E-10 | 0.0336 | 0.0132 | 0.0112 |
| rs34975158 | 6 | 32886487 | A | G | 0.068725 | 63.43161 | -0.5145 | 0.0646 | 1.73E-15 | -0.0127 | 0.0297 | 0.668099 |
| rs373126870 | 6 | 28590786 | G | C | 0.035668 | 31.24556 | -0.4919 | 0.088 | 2.28E-08 | -0.0283 | 0.0274 | 0.3007 |
| rs3998115 | 6 | 33784833 | C | G | 0.704906 | 71.10938 | 0.4444 | 0.0527 | 3.54E-17 | 0.0009 | 0.0142 | 0.9498 |
| rs6913309 | 6 | 32339840 | A | T | 0.229264 | 117.3216 | -0.495 | 0.0457 | 2.53E-27 | -0.0316 | 0.0204 | 0.1205 |
| rs72661136 | 6 | 29622516 | C | T | 0.018097 | 38.10631 | -0.5173 | 0.0838 | 6.77E-10 | -0.1282 | 0.0568 | 0.02391 |
| rs73739611 | 6 | 33033188 | T | C | 0.604606 | 192.3078 | 0.4396 | 0.0317 | 8.21E-44 | 0.0259 | 0.0209 | 0.2168 |
| rs7741871 | 6 | 33864354 | A | G | 0.228037 | 56.83327 | -0.3596 | 0.0477 | 4.76E-14 | -0.0014 | 0.0136 | 0.921 |
| rs79690458 | 6 | 32140848 | T | A | 0.060488 | 60.43809 | -0.5302 | 0.0682 | 7.44E-15 | 0.0202 | 0.0336 | 0.547299 |
| rs9277665 | 6 | 33085462 | A | G | 0.222179 | 41.85934 | -0.2148 | 0.0332 | 1.01E-10 | -0.0063 | 0.0161 | 0.696899 |

**Supplementary Table 2 Mendelian randomization analysis of chronic hepatitis B and coronary heart disease.**

| SNP | Chr | Position | A1 | A2 | EAF | F | Chronic hepatitis B | | | Coronary heart disease | | |
| --- | --- | --- | --- | --- | --- | --- | --- | --- | --- | --- | --- | --- |
|  |  |  |  |  |  |  | **Beta** | **SE** | **P-val** | **Beta** | **SE** | **P-val** |
| rs114484678 | 6 | 32215057 | C | T | 0.063896 | 40.15369 | -0.3143 | 0.0496 | 2.33E-10 | 0.0631 | 0.0263 | 0.01654 |
| rs115552552 | 6 | 33346116 | T | C | 0.039869 | 50.55374 | -0.5553 | 0.0781 | 1.13E-12 | -0.0423 | 0.0342 | 0.2163 |
| rs115888238 | 6 | 32967965 | C | G | 0.077911 | 80.96698 | -0.4904 | 0.0545 | 2.17E-19 | -0.0024 | 0.0303 | 0.9377 |
| rs11754012 | 6 | 33597696 | T | C | 0.138049 | 77.13696 | -0.4892 | 0.0557 | 1.62E-18 | -0.0171 | 0.0212 | 0.4201 |
| rs117810449 | 6 | 32445700 | C | T | 0.054877 | 46.10614 | 0.3171 | 0.0467 | 1.16E-11 | 0.0026 | 0.039 | 0.9459 |
| rs12214820 | 6 | 27537104 | A | G | 0.123769 | 37.77547 | -0.4499 | 0.0732 | 7.97E-10 | -0.024 | 0.0176 | 0.1722 |
| rs148198842 | 6 | 30690169 | A | G | 0.024112 | 49.94494 | -0.5576 | 0.0789 | 1.55E-12 | -0.0172 | 0.0573 | 0.764599 |
| rs16870693 | 6 | 32711691 | A | C | 0.060517 | 43.00333 | 0.2728 | 0.0416 | 5.55E-11 | -0.1627 | 0.2897 | 0.5745 |
| rs17200976 | 6 | 31674996 | G | A | 0.031991 | 40.89907 | -0.4029 | 0.063 | 1.59E-10 | -0.2222 | 0.1275 | 0.0815 |
| rs2021835 | 6 | 27778097 | T | C | 0.039978 | 30.40279 | -0.4571 | 0.0829 | 3.47E-08 | -0.065 | 0.0285 | 0.0227 |
| rs3131004 | 6 | 31095294 | G | A | 0.568226 | 41.44948 | 0.1925 | 0.0299 | 1.16E-10 | 0.0318 | 0.0136 | 0.01955 |
| rs34975158 | 6 | 32886487 | A | G | 0.068725 | 63.43161 | -0.5145 | 0.0646 | 1.73E-15 | 0.0128 | 0.0305 | 0.6743 |
| rs373126870 | 6 | 28590786 | G | C | 0.035668 | 31.24556 | -0.4919 | 0.088 | 2.28E-08 | -0.0297 | 0.0282 | 0.2929 |
| rs3998115 | 6 | 33784833 | C | G | 0.704906 | 71.10938 | 0.4444 | 0.0527 | 3.54E-17 | 0.0069 | 0.0146 | 0.6362 |
| rs6913309 | 6 | 32339840 | A | T | 0.229264 | 117.3216 | -0.495 | 0.0457 | 2.53E-27 | -0.0666 | 0.021 | 0.001498 |
| rs72661136 | 6 | 29622516 | C | T | 0.018097 | 38.10631 | -0.5173 | 0.0838 | 6.77E-10 | -0.0783 | 0.0579 | 0.1768 |
| rs73739611 | 6 | 33033188 | T | C | 0.604606 | 192.3078 | 0.4396 | 0.0317 | 8.21E-44 | 0.0016 | 0.0216 | 0.9402 |
| rs7741871 | 6 | 33864354 | A | G | 0.228037 | 56.83327 | -0.3596 | 0.0477 | 4.76E-14 | -0.0032 | 0.014 | 0.817 |
| rs79690458 | 6 | 32140848 | T | A | 0.060488 | 60.43809 | -0.5302 | 0.0682 | 7.44E-15 | 0.0475 | 0.0348 | 0.1719 |
| rs9277665 | 6 | 33085462 | A | G | 0.222179 | 41.85934 | -0.2148 | 0.0332 | 1.01E-10 | -0.0108 | 0.0165 | 0.5121 |

**Supplementary Table 3 Mendelian randomization analysis of chronic hepatitis B and hypertension.**

| SNP | Chr | Position | A1 | A2 | EAF | F | Chronic hepatitis B | | | Hypertension | | |
| --- | --- | --- | --- | --- | --- | --- | --- | --- | --- | --- | --- | --- |
|  |  |  |  |  |  |  | **Beta** | **SE** | **P-val** | **Beta** | **SE** | **P-val** |
| rs114484678 | 6 | 32215057 | C | T | 0.063896 | 40.15369 | -0.3143 | 0.0496 | 2.33E-10 | 0.0109 | 0.0185 | 0.557 |
| rs115552552 | 6 | 33346116 | T | C | 0.039869 | 50.55374 | -0.5553 | 0.0781 | 1.13E-12 | -0.0044 | 0.0242 | 0.8549 |
| rs115888238 | 6 | 32967965 | C | G | 0.077911 | 80.96698 | -0.4904 | 0.0545 | 2.17E-19 | -0.0419 | 0.0214 | 0.05067 |
| rs11754012 | 6 | 33597696 | T | C | 0.138049 | 77.13696 | -0.4892 | 0.0557 | 1.62E-18 | -0.0025 | 0.015 | 0.866 |
| rs117810449 | 6 | 32445700 | C | T | 0.054877 | 46.10614 | 0.3171 | 0.0467 | 1.16E-11 | 0.0108 | 0.0274 | 0.694201 |
| rs12214820 | 6 | 27537104 | A | G | 0.123769 | 37.77547 | -0.4499 | 0.0732 | 7.97E-10 | -0.0093 | 0.0124 | 0.4548 |
| rs148198842 | 6 | 30690169 | A | G | 0.024112 | 49.94494 | -0.5576 | 0.0789 | 1.55E-12 | -0.0972 | 0.0404 | 0.01607 |
| rs16870693 | 6 | 32711691 | A | C | 0.060517 | 43.00333 | 0.2728 | 0.0416 | 5.55E-11 | -0.2834 | 0.1931 | 0.1422 |
| rs17200976 | 6 | 31674996 | G | A | 0.031991 | 40.89907 | -0.4029 | 0.063 | 1.59E-10 | -0.021 | 0.0887 | 0.8126 |
| rs2021835 | 6 | 27778097 | T | C | 0.039978 | 30.40279 | -0.4571 | 0.0829 | 3.47E-08 | 0.0096 | 0.0201 | 0.634 |
| rs3131004 | 6 | 31095294 | G | A | 0.568226 | 41.44948 | 0.1925 | 0.0299 | 1.16E-10 | 0.042 | 0.0096 | 1.3E-05 |
| rs34975158 | 6 | 32886487 | A | G | 0.068725 | 63.43161 | -0.5145 | 0.0646 | 1.73E-15 | -0.0031 | 0.0217 | 0.8862 |
| rs373126870 | 6 | 28590786 | G | C | 0.035668 | 31.24556 | -0.4919 | 0.088 | 2.28E-08 | 0.0001 | 0.0198 | 0.9946 |
| rs3998115 | 6 | 33784833 | C | G | 0.704906 | 71.10938 | 0.4444 | 0.0527 | 3.54E-17 | 0.0246 | 0.0103 | 0.01685 |
| rs6913309 | 6 | 32339840 | A | T | 0.229264 | 117.3216 | -0.495 | 0.0457 | 2.53E-27 | -0.0302 | 0.0148 | 0.04188 |
| rs72661136 | 6 | 29622516 | C | T | 0.018097 | 38.10631 | -0.5173 | 0.0838 | 6.77E-10 | -0.0408 | 0.041 | 0.3189 |
| rs73739611 | 6 | 33033188 | T | C | 0.604606 | 192.3078 | 0.4396 | 0.0317 | 8.21E-44 | -0.0274 | 0.0152 | 0.07095 |
| rs7741871 | 6 | 33864354 | A | G | 0.228037 | 56.83327 | -0.3596 | 0.0477 | 4.76E-14 | 0.0062 | 0.0099 | 0.5334 |
| rs79690458 | 6 | 32140848 | T | A | 0.060488 | 60.43809 | -0.5302 | 0.0682 | 7.44E-15 | 0.0346 | 0.0244 | 0.1563 |
| rs9277665 | 6 | 33085462 | A | G | 0.222179 | 41.85934 | -0.2148 | 0.0332 | 1.01E-10 | 0.0042 | 0.0117 | 0.7217 |

**Supplemental Table 4 Mendelian randomization analysis of chronic hepatitis B and ischemic stroke.**

| SNP | Chr | Position | A1 | A2 | EAF | F | Chronic hepatitis B | | | Ischemic stroke | | |
| --- | --- | --- | --- | --- | --- | --- | --- | --- | --- | --- | --- | --- |
|  |  |  |  |  |  |  | **Beta** | **SE** | **P-val** | **Beta** | **SE** | **P-val** |
| rs1005599 | 6 | 31276447 | C | G | 0.747166 | 68.5791 | 0.371 | 0.0448 | 1.21E-16 | -0.0297 | 0.0129 | 0.02157 |
| rs114484678 | 6 | 32215057 | C | T | 0.063896 | 40.15369 | -0.3143 | 0.0496 | 2.33E-10 | 0.0277 | 0.0149 | 0.062589 |
| rs115552552 | 6 | 33346116 | T | C | 0.039869 | 50.55374 | -0.5553 | 0.0781 | 1.13E-12 | -0.0124 | 0.0213 | 0.560899 |
| rs115888238 | 6 | 32967965 | C | G | 0.077911 | 80.96698 | -0.4904 | 0.0545 | 2.17E-19 | -0.0071 | 0.0155 | 0.649299 |
| rs11754012 | 6 | 33597696 | T | C | 0.138049 | 77.13696 | -0.4892 | 0.0557 | 1.62E-18 | 0.0165 | 0.014 | 0.2363 |
| rs117810449 | 6 | 32445700 | C | T | 0.054877 | 46.10614 | 0.3171 | 0.0467 | 1.16E-11 | -0.0203 | 0.0154 | 0.187 |
| rs12214820 | 6 | 27537104 | A | G | 0.123769 | 37.77547 | -0.4499 | 0.0732 | 7.97E-10 | 0.0047 | 0.0155 | 0.7602 |
| rs148198842 | 6 | 30690169 | A | G | 0.024112 | 49.94494 | -0.5576 | 0.0789 | 1.55E-12 | 0.0329 | 0.0242 | 0.174 |
| rs16870693 | 6 | 32711691 | A | C | 0.060517 | 43.00333 | 0.2728 | 0.0416 | 5.55E-11 | -0.0225 | 0.0143 | 0.1153 |
| rs17200976 | 6 | 31674996 | G | A | 0.031991 | 40.89907 | -0.4029 | 0.063 | 1.59E-10 | 0.0072 | 0.0204 | 0.723601 |
| rs201417696 | 6 | 30312962 | G | T | 0.135228 | 37.01468 | -0.3188 | 0.0524 | 1.21E-09 | 0.0111 | 0.018 | 0.5358 |
| rs2021835 | 6 | 27778097 | T | C | 0.039978 | 30.40279 | -0.4571 | 0.0829 | 3.47E-08 | 0.0195 | 0.0215 | 0.3625 |
| rs28715762 | 6 | 32619578 | C | A | 0.084693 | 67.92218 | -0.5085 | 0.0617 | 1.65E-16 | 0.0208 | 0.0204 | 0.3076 |
| rs28746784 | 6 | 32632261 | T | C | 0.301764 | 143.2268 | 0.4452 | 0.0372 | 6.05E-33 | -0.0295 | 0.0128 | 0.02074 |
| rs28826696 | 6 | 32617628 | C | T | 0.742601 | 181.904 | 0.526 | 0.039 | 1.5E-41 | -0.0484 | 0.013 | 0.000202 |
| rs3131004 | 6 | 31095294 | G | A | 0.568226 | 41.44948 | 0.1925 | 0.0299 | 1.16E-10 | -0.0082 | 0.0086 | 0.3394 |
| rs34975158 | 6 | 32853511 | A | G | 0.068725 | 63.43161 | -0.5145 | 0.0646 | 1.73E-15 | 0.0167 | 0.0191 | 0.3832 |
| rs373126870 | 6 | 28590786 | G | C | 0.035668 | 31.24556 | -0.4919 | 0.088 | 2.28E-08 | 0.0177 | 0.0246 | 0.4708 |
| rs3998115 | 6 | 33784833 | C | G | 0.704906 | 71.10938 | 0.4444 | 0.0527 | 3.54E-17 | -0.0191 | 0.0116 | 0.099749 |
| rs6913309 | 6 | 32339840 | A | T | 0.229264 | 117.3216 | -0.495 | 0.0457 | 2.53E-27 | 0.0066 | 0.0119 | 0.577301 |
| rs72661136 | 6 | 29622516 | C | T | 0.018097 | 38.10631 | -0.5173 | 0.0838 | 6.77E-10 | 0.0262 | 0.0259 | 0.3113 |
| rs73739611 | 6 | 33033188 | T | C | 0.604606 | 192.3078 | 0.4396 | 0.0317 | 8.21E-44 | 0.0147 | 0.0103 | 0.1533 |
| rs7741871 | 6 | 33864354 | A | G | 0.228037 | 56.83327 | -0.3596 | 0.0477 | 4.76E-14 | -0.0117 | 0.0114 | 0.3028 |
| rs79690458 | 6 | 32140848 | T | A | 0.060488 | 60.43809 | -0.5302 | 0.0682 | 7.44E-15 | 0.0132 | 0.0189 | 0.4849 |
| rs9276410 | 6 | 32710396 | A | G | 0.622217 | 32.67091 | 0.2092 | 0.0366 | 1.06E-08 | 0.0002 | 0.0112 | 0.9887 |
| rs9277665 | 6 | 33085462 | A | G | 0.222179 | 41.85934 | -0.2148 | 0.0332 | 1.01E-10 | -0.0087 | 0.0098 | 0.3723 |

## Supplementary Figures


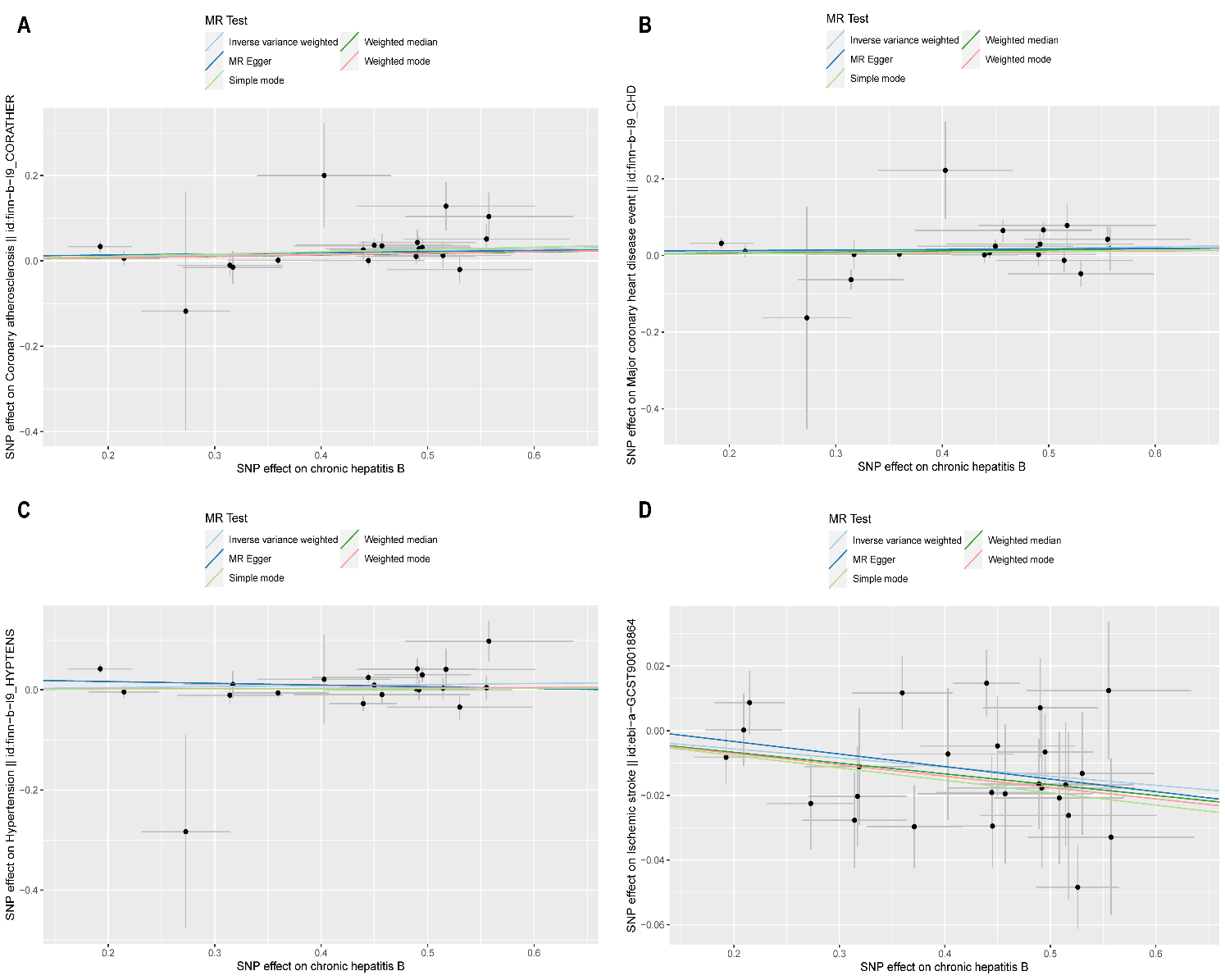


Supplementary Figure 1 Scatter plot: (A) CHB and Atherosclerosis; (B) CHB and CHD; (C) CHB and Hypertension; (D) CHB and IS


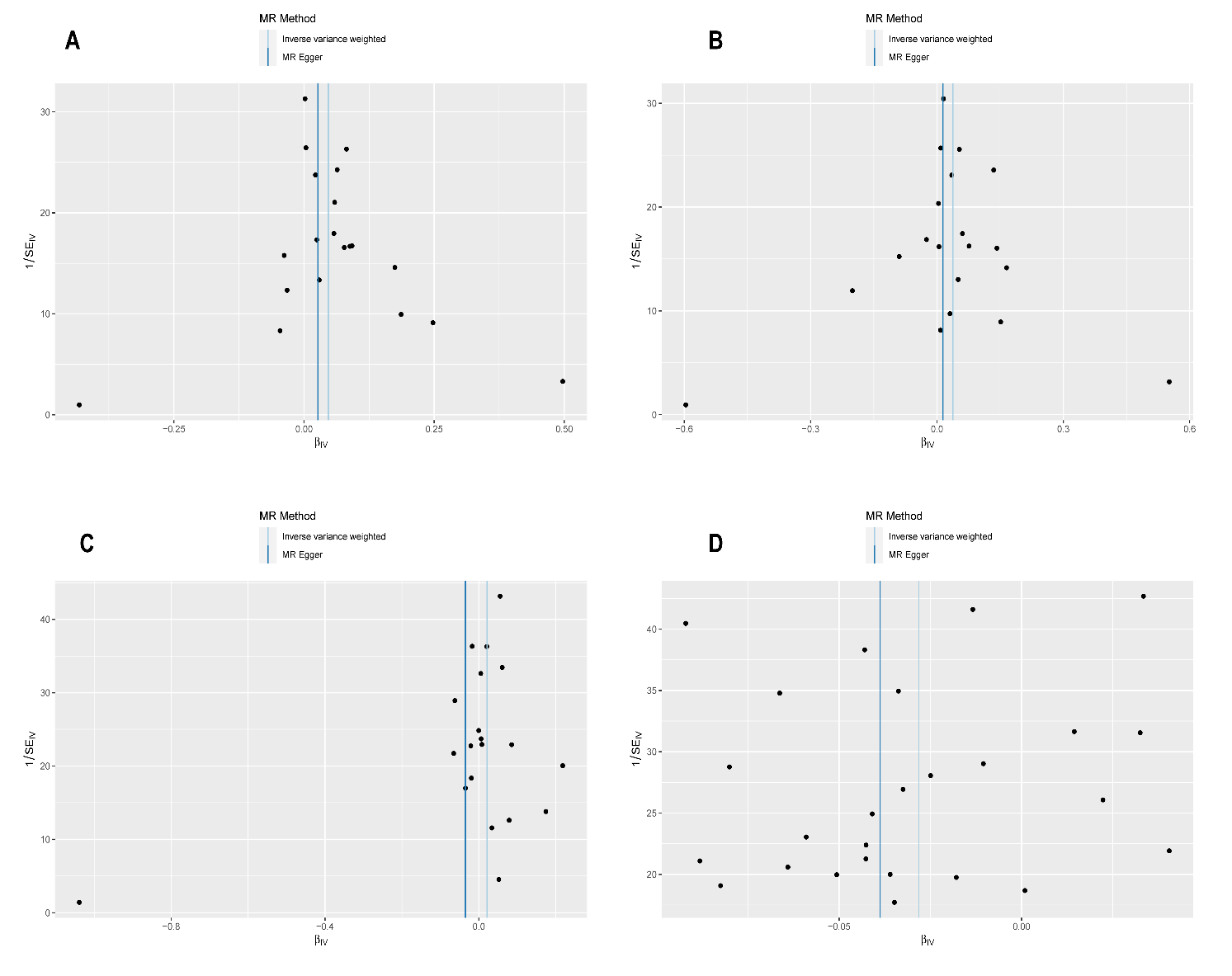


Supplementary Figure 2 Funnel plot: (A) CHB and Atherosclerosis; (B) CHB and CHD; (C) CHB and Hypertension; (D) CHB and IS
